# Supplementary material for: A High-Density Linkage Map Reveals Sexual Dimorphism in Recombination Landscapes in Red Deer (Cervus elaphus)
Source: G3 (Bethesda). 2017 Jun 30;7(8):2859–70. doi: 10.1534/g3.117.044198 (PMC5555489; doi:10.1534/g3.117.044198)
Supplement: Supplementary file 1 [file 2859FileS1.pdf]

Supplementary Figures

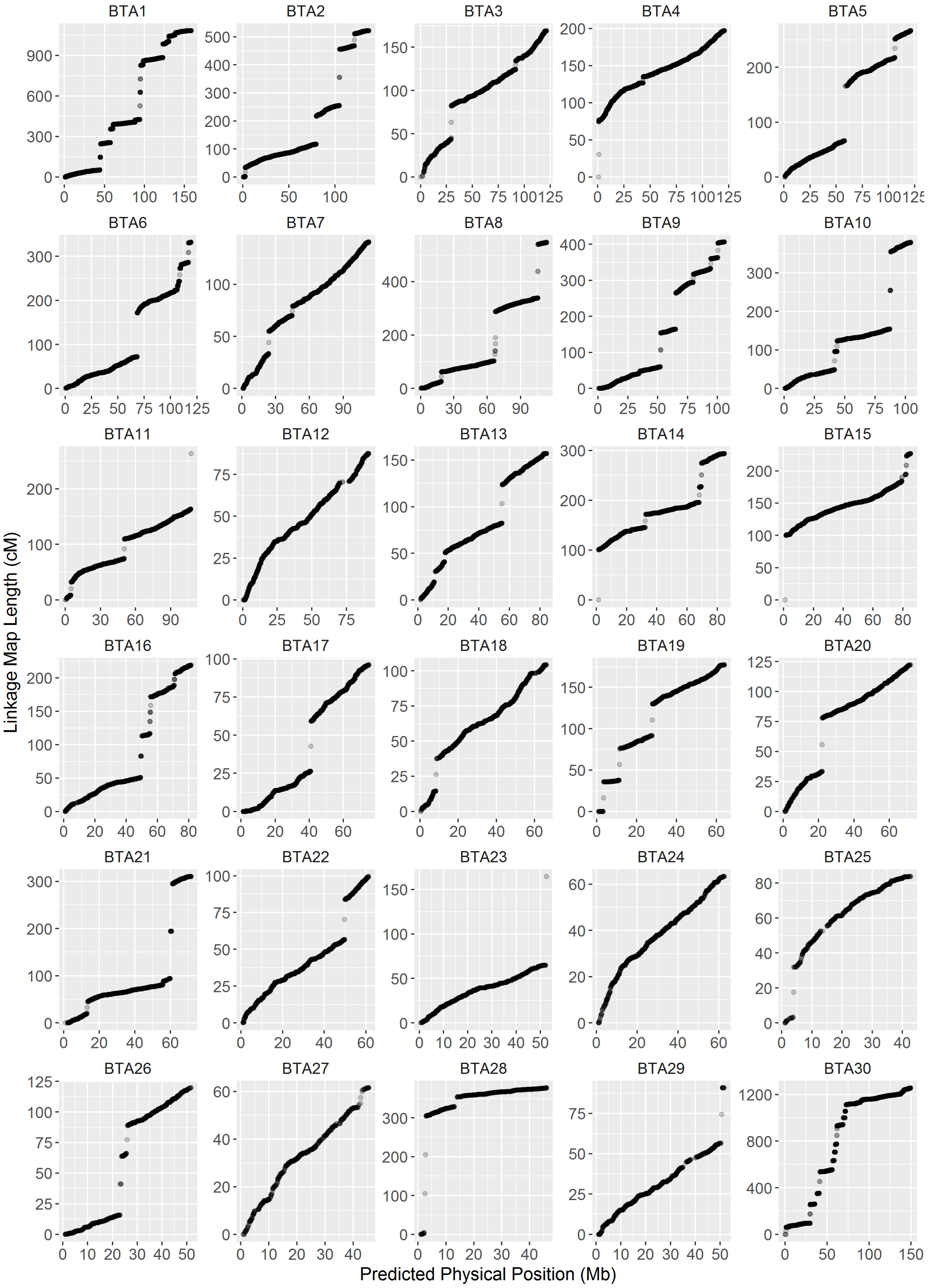

Figure S1: Build 1: Sex-averaged linkage maps assuming complete synteny of chromosomes and locus positions with the cattle genome. The x-axis gives the predicted cattle positions, and the y-axis gives the linkage map positions.

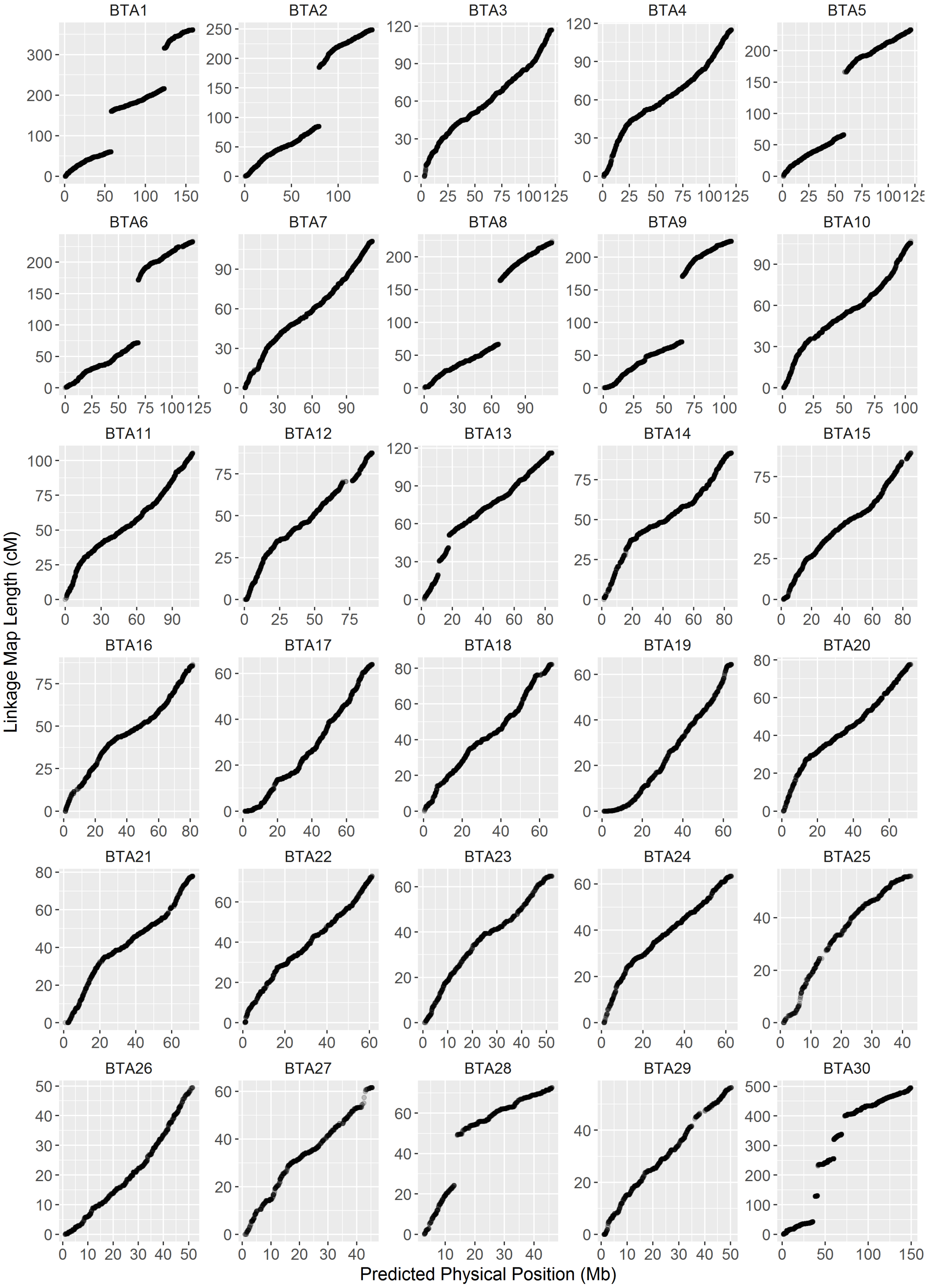

Figure S2: Build 2: Sex-averaged linkage maps after removing SNPs that were predicted to be wrongly mapped. Chromosome numbers are based on synteny with the cattle genome. The x-axis gives the predicted cattle positions, and the y-axis gives the linkage map positions.

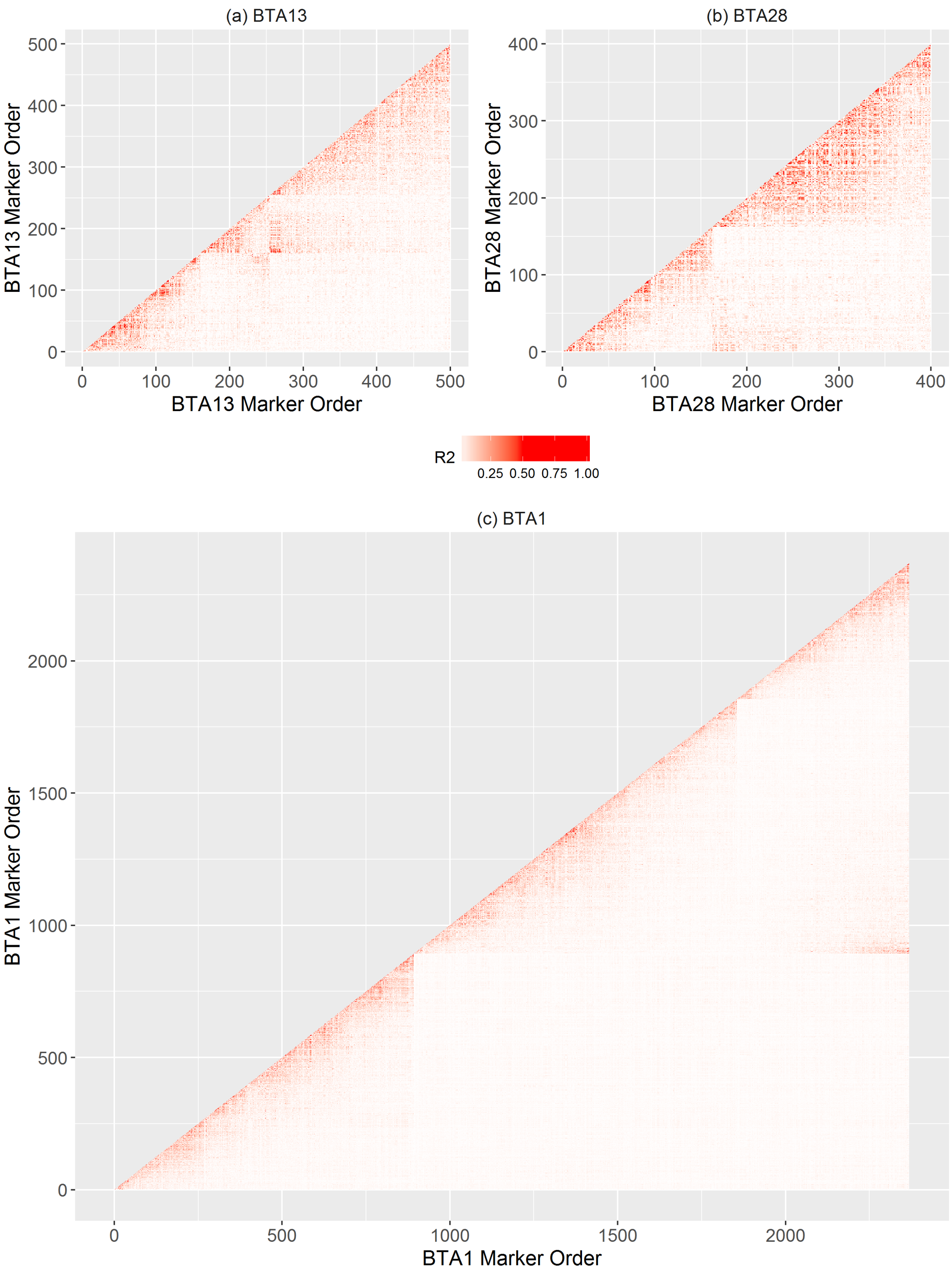

Figure S3: Patterns of LD (R^2) on (a) BTA13, (b) BTA28 and (c) all SNPs on BTA1.

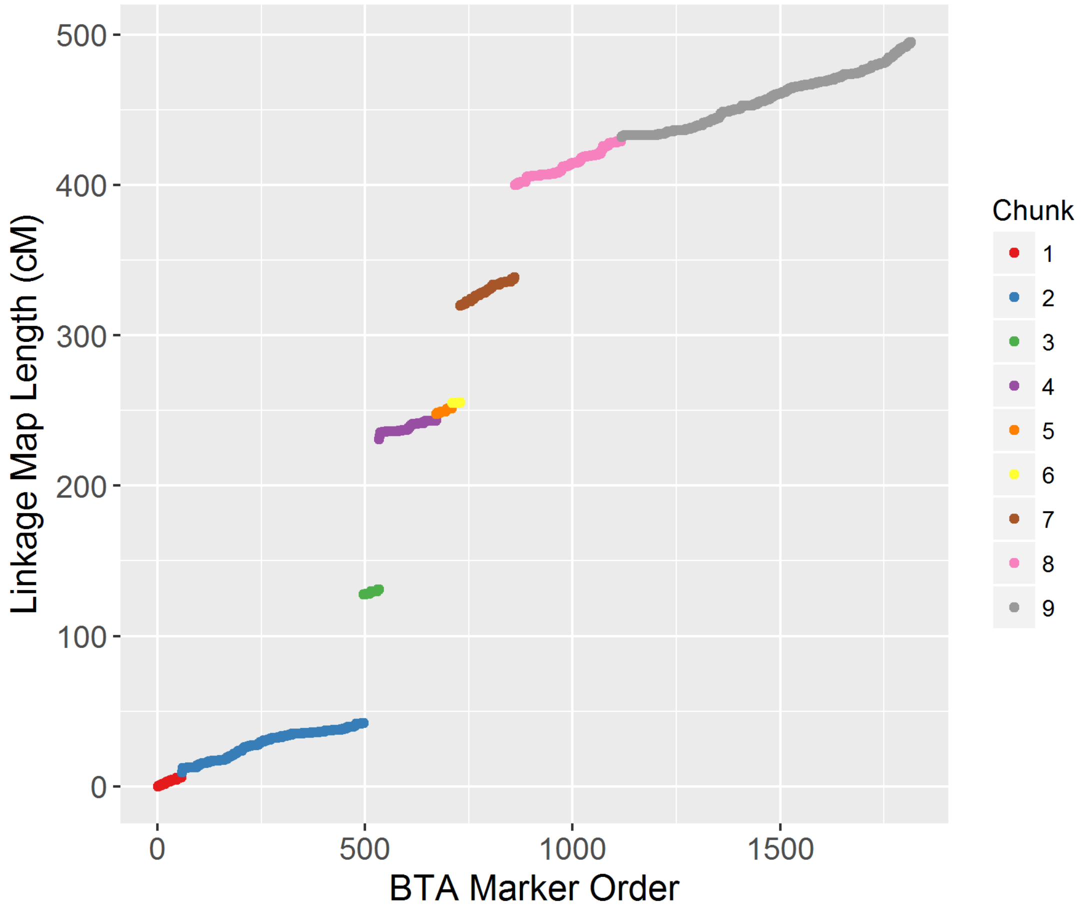

Figure S4: Marker order and linkage map distance on BTA30 (CEL34) after Build 2. Colours indicate chunks flanked by recombination fractions of $\geq$ 3cM.

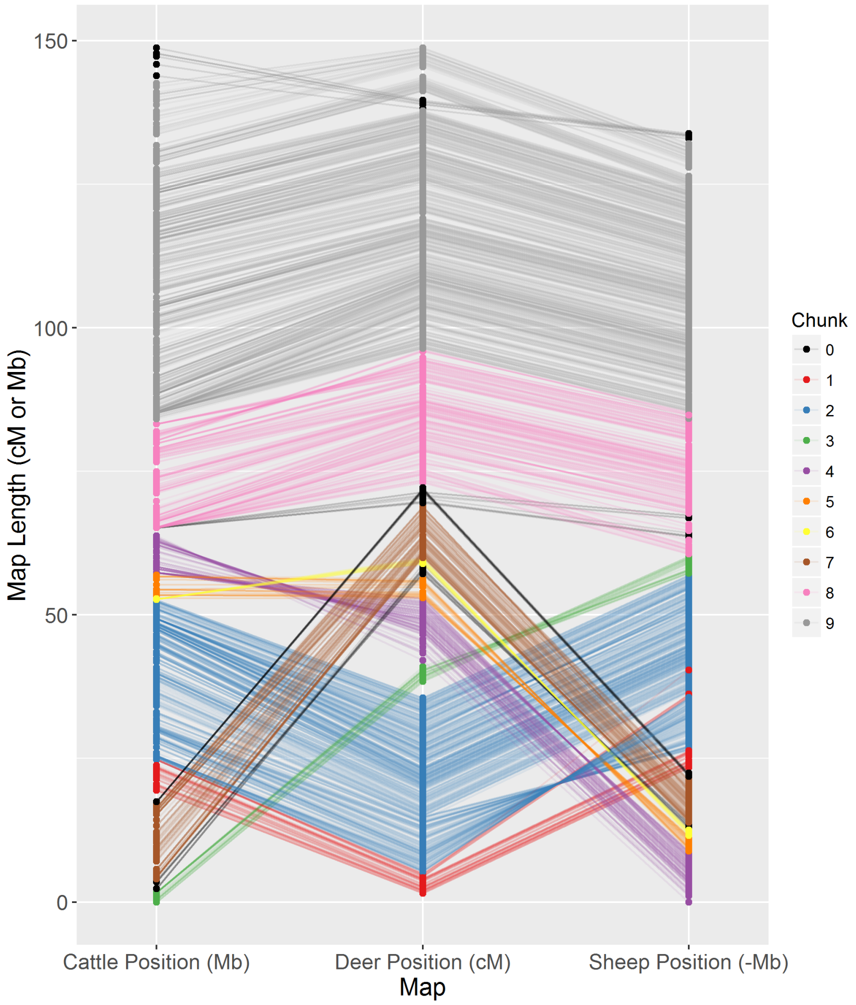

Figure S5: Comparison of map positions on the X chromosome between cattle (BTA30, distance in Mb from genome build BTA vUMD 3.0), deer (CEL34, distance in cM from Build 5) and sheep (OAR27, distance in Mb and reversed from genome build Oar_v3.1). Colours indicate chunks flanked by recombination fractions of $\geq$ 3cM in Build 2. Chunk 0 indicates markers unmapped in Build 2 that were retrospectively added to Build 5. Full data for this figure is provided in Table S2.

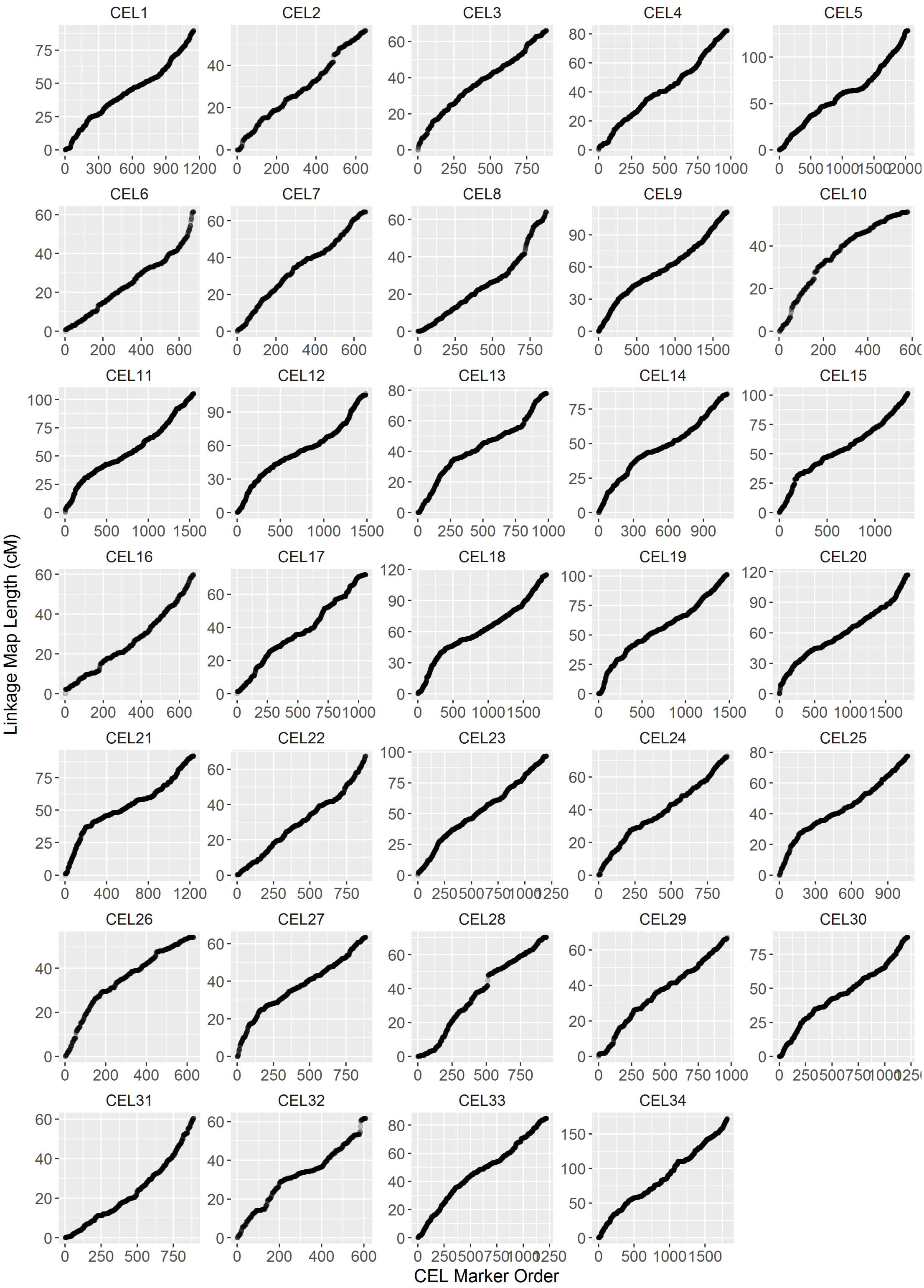

Figure S6: Build 3: Sex-averaged linkage maps after carrying out chromosomal re-arrangements outlined in the main text. Chromosome numbers are *Cervus elaphus* (CEL) linkage groups assuming grouping as in Slate et al (Slate *et al.*, 2002) (Table 1). The x-axis gives the predicted order of the deer loci on the linkage groups after Build 3.

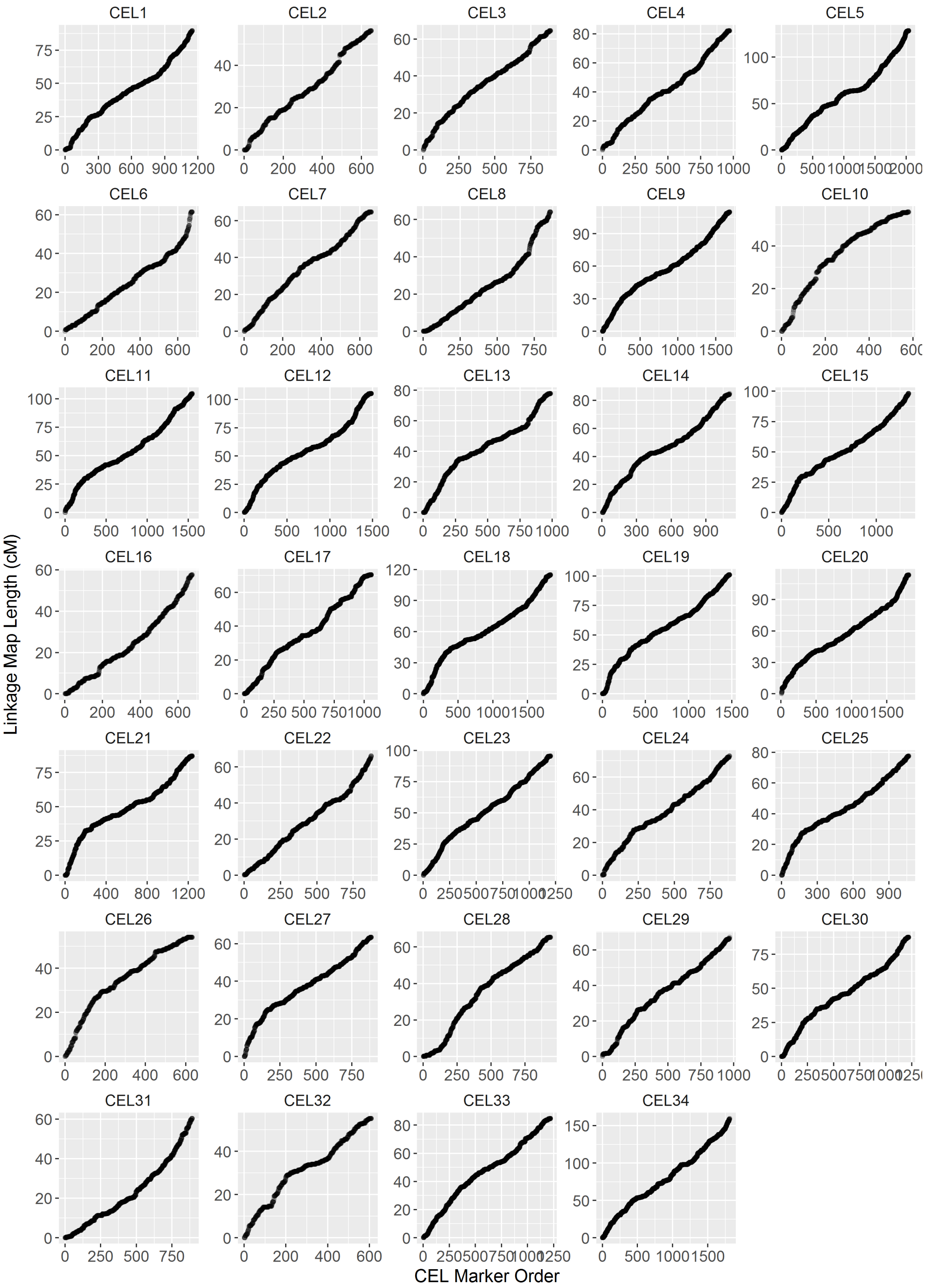

Figure S7: Build 4: Sex-averaged linkage maps after testing inversion and deletion of short chunks as outlined in the main text. Chromosome numbers are *Cervus elaphus* (CEL) linkage groups. The x-axis gives the predicted order of the deer loci on the linkage groups after Build 4.

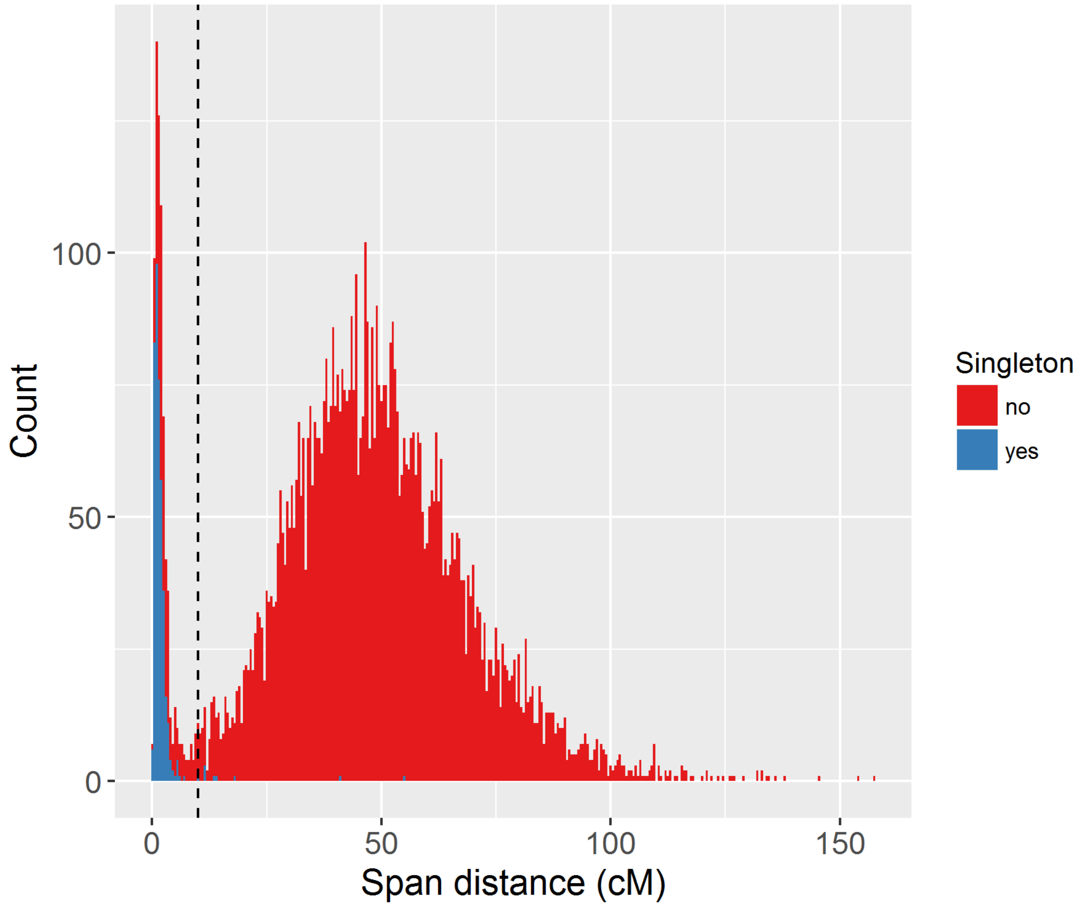

Figure S8: Histogram of the span distances (in Mb) between double crossovers on autosomal chromatids. Bar segments are colour coded as double crossovers spanning a single SNP locus (blue) and those spanning more than one SNP (red). All double crossovers across a single SNP were discarded from the dataset, as they are likely to be the result of a genotyping error at that SNP. Short crossovers below a span distance of 10cM were also discarded (see text for rationale)

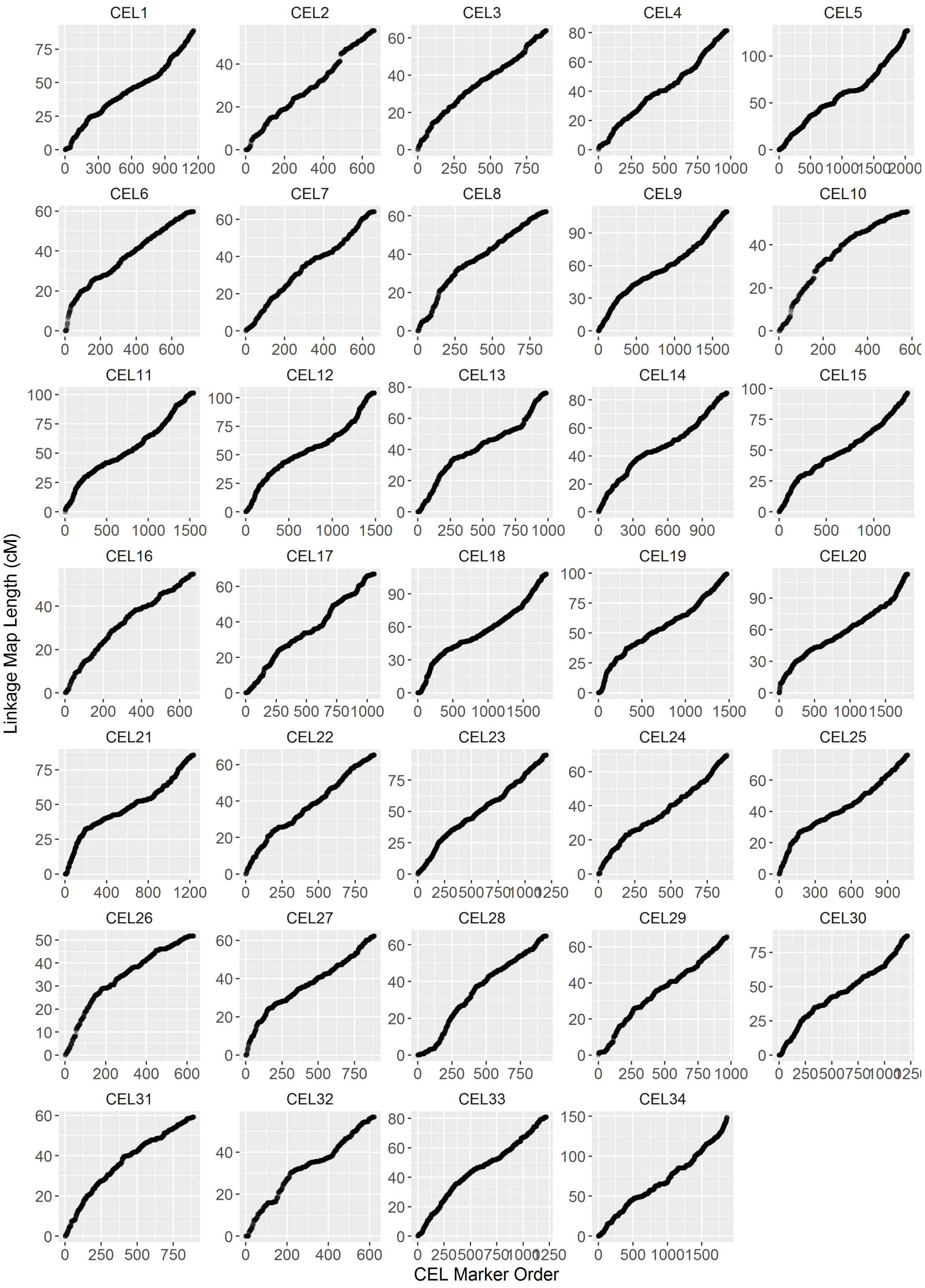

Figure S9: Build 5: Sex-averaged linkage maps after removing incorrectly-called double crossovers. Chromosome numbers are *Cervus elaphus* (CEL) linkage groups. The x-axis gives the predicted order of the deer loci on the linkage groups after Build 4, and the y-axis gives the linkage map positions.

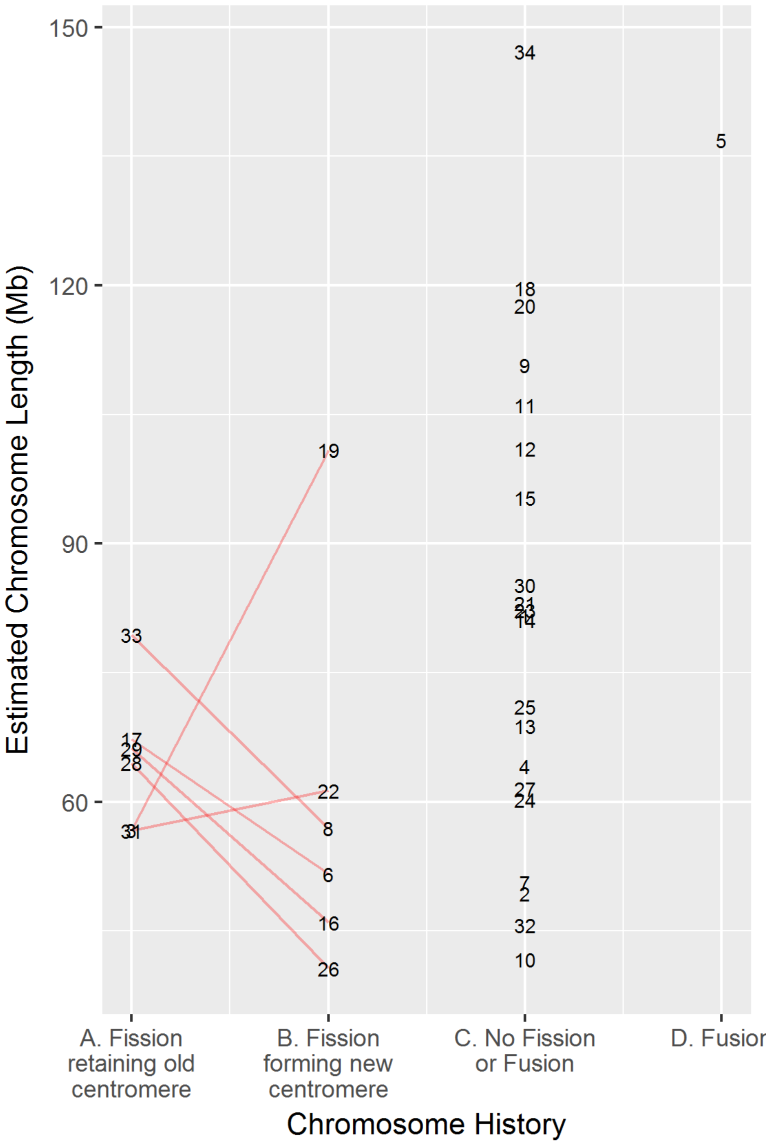

Figure S10: Estimated chromosome physical length (Mb) for different chromosome histories. Numbers indicate the linkage group; some have been jittered horizontally to allow easier reading. Red lines connect fission chromosomes that had a common origin.

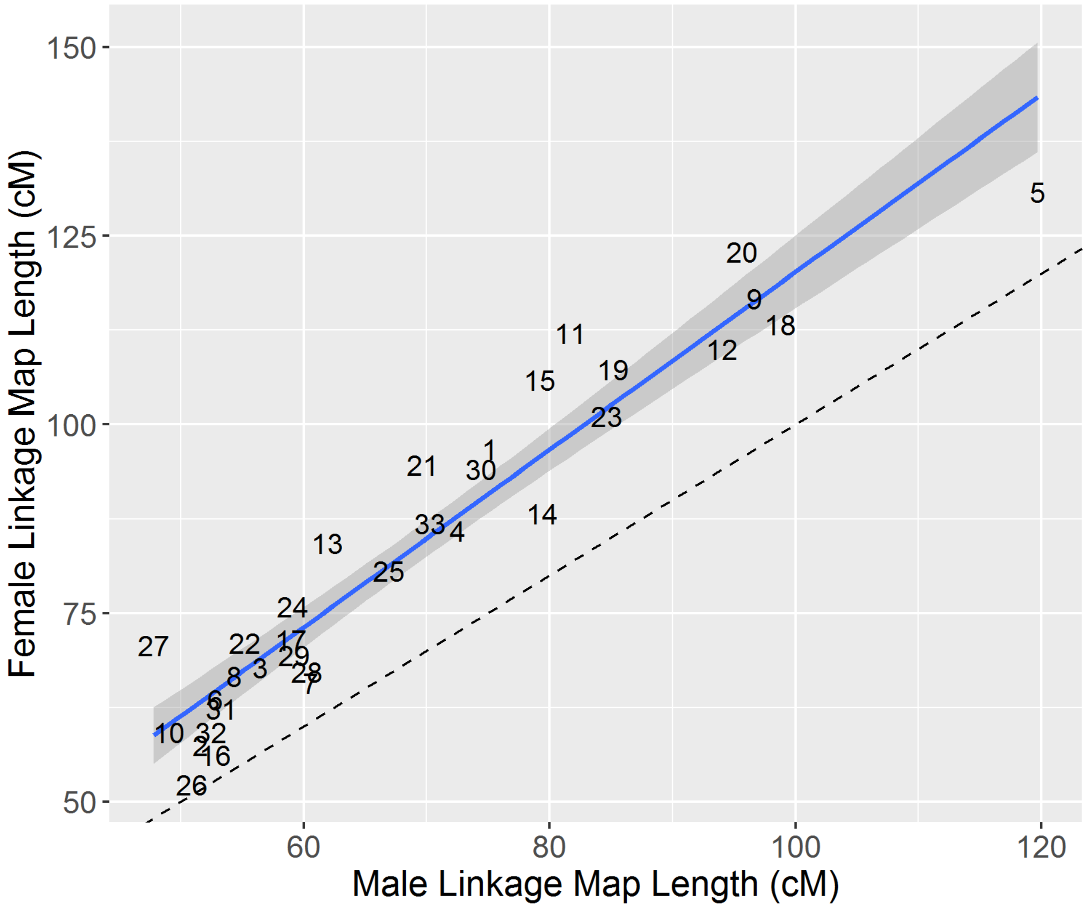

Figure S11: Correlation between male and female linkage map lengths (cM). The line and the gray-shaded area indicates the regression slope and standard error, respectively. The dashed line in C indicates where male and female linkage maps would be of equal length.

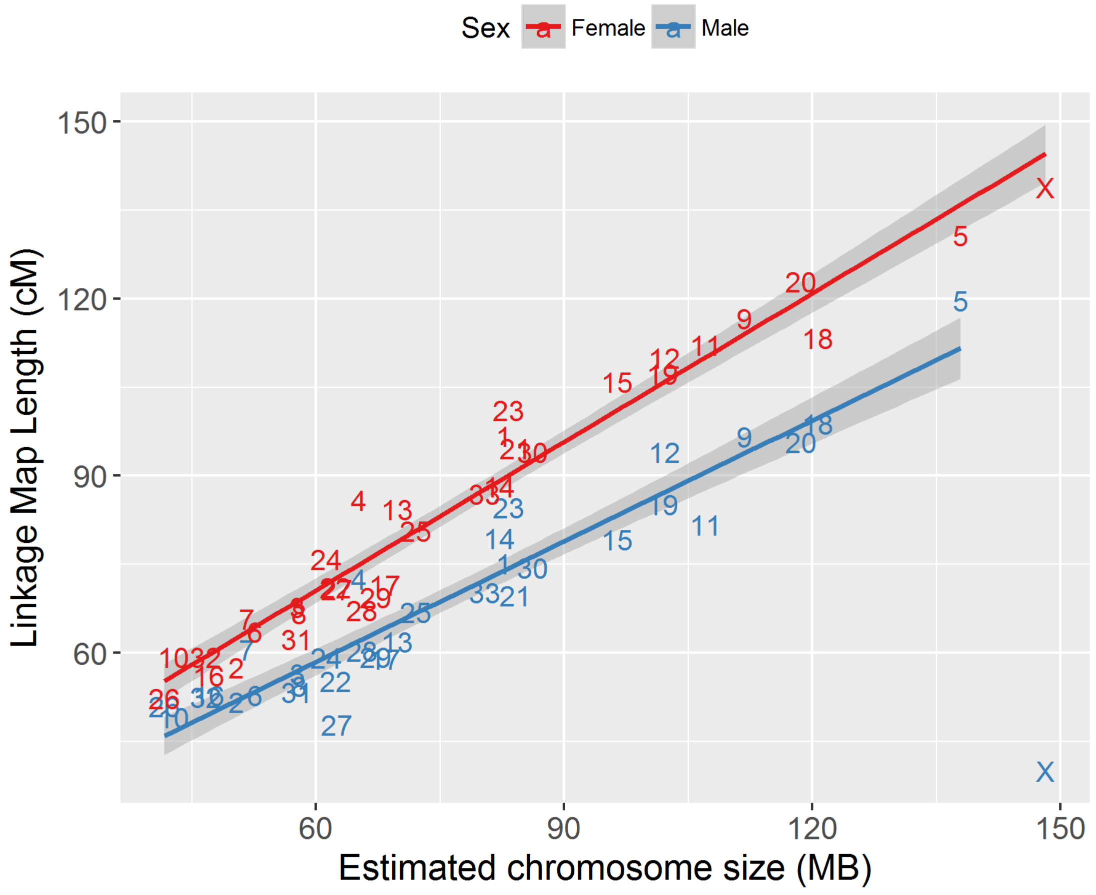

Figure S12: Sex-specific correlations between male and female linkage map lengths (cM). The line and the gray-shaded area indicates the regression slope and standard error, respectively.

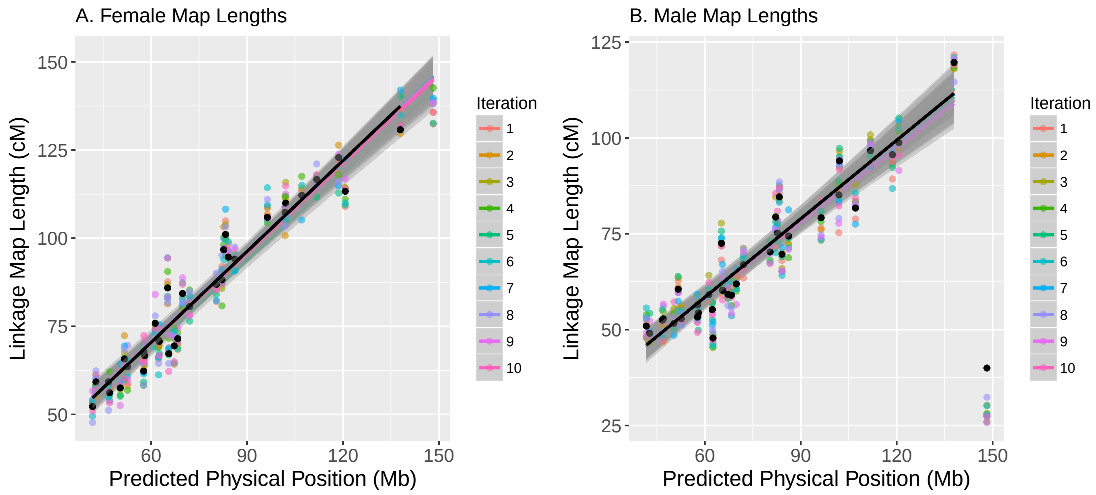

Figure S13: Correlations between the predicted physical position (Mb) and linkage map lengths (cM) for ten subsets of 483 females (A) and males (B) randomly sampled with replacement (coloured lines) and as observed in the data (black line).

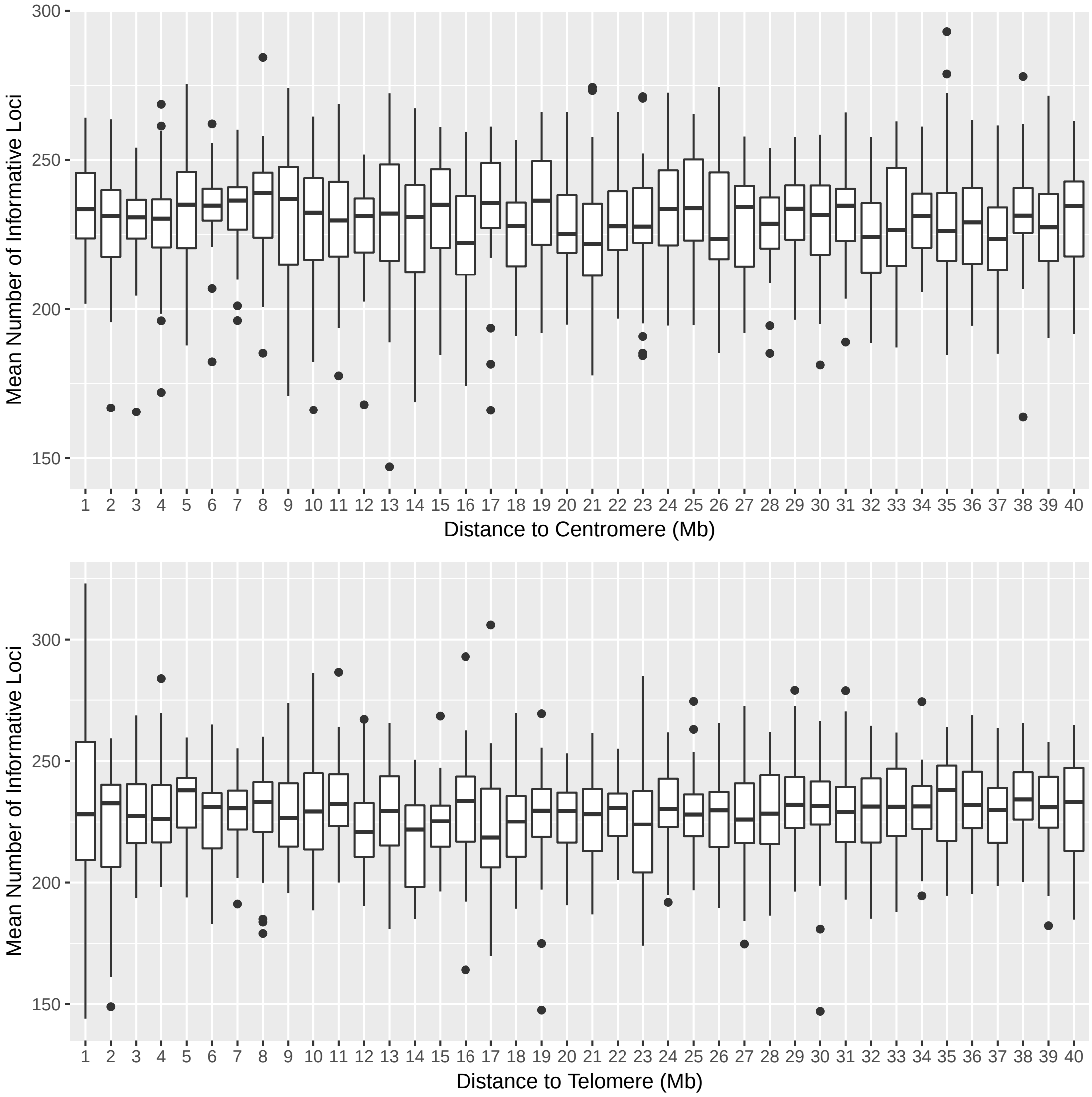

Figure S14: Mean number of informative loci within each 1Mb window across acrocentric autosomes. The top panel shows window proximity to the centromere; the lower panel shows window proximity to the telomere. Large variance in the first window relative to the telomere is likely to be due to the variable size of this last segment (i.e. <1Mb is characterised)

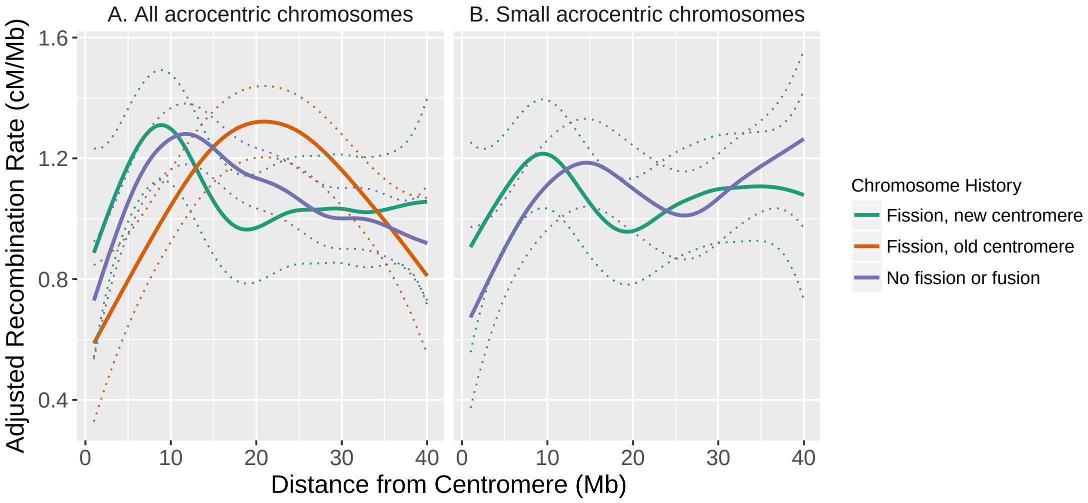

Figure S15: General additive model curves of adjusted recombination rate in females (k = 10). A. All acrocentric chromosomes, including fission chromosomes forming a new centromere (n = 6), fission chromosomes retaining the existing centromere (n = 6) and chromosomes with no fission or fusion (n = 20). B. Small acrocentric chromosomes, including fission chromosomes forming a new centromere (n = 5) and chromosomes with no fission or fusion (n = 6). Dashed lines indicate the standard errors. Recombination rates were adjusted for chromosome length (see main text).

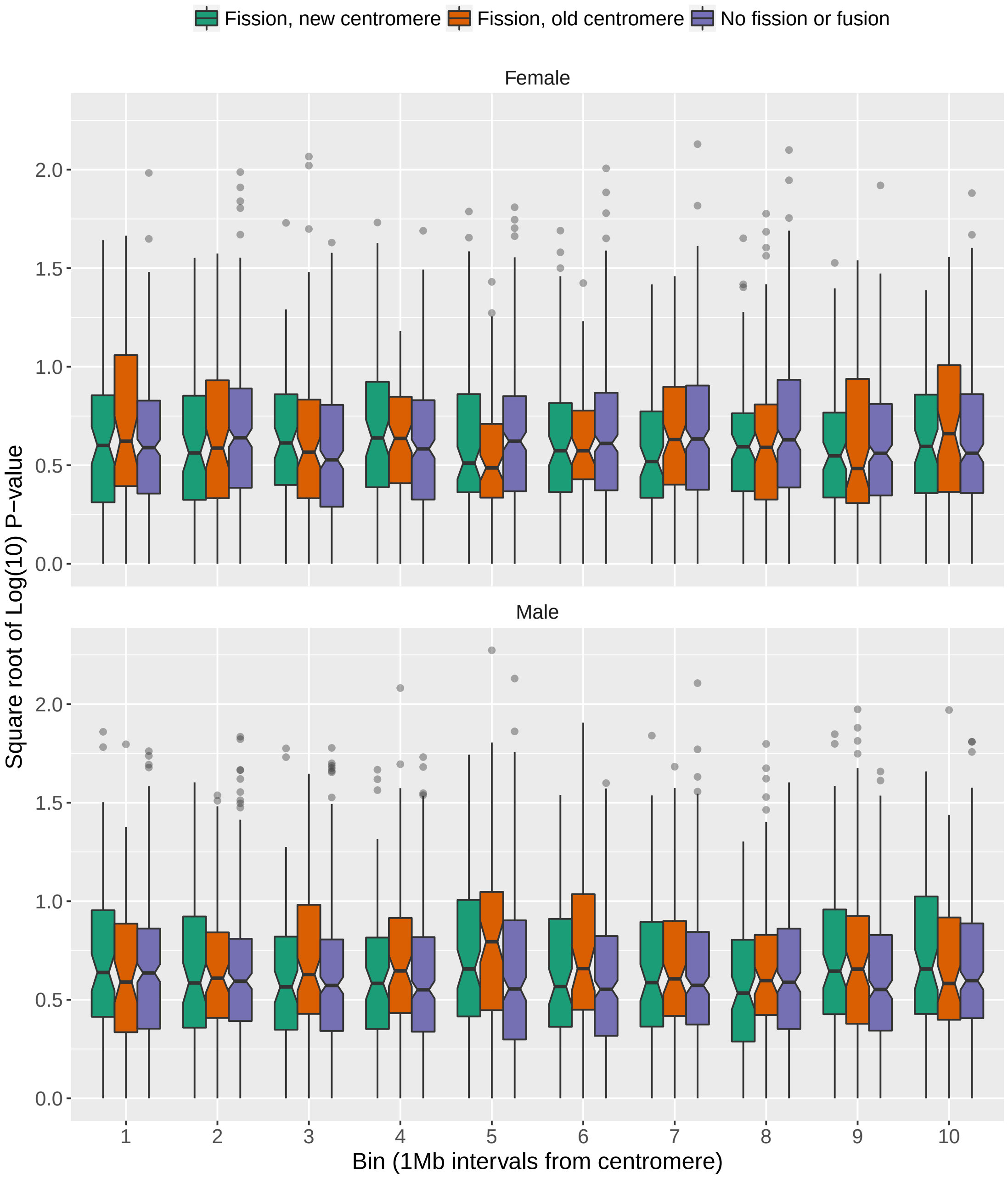

Figure S16: Boxplot of transformed P-values for transmission distortion in 1Mb intervals for acrocentric chromosomes of different histories in females and males.

Supplementary Tables

Supplementary tables are available online and in the data archive: doi:10.6084/m9.figshare.5002562.

Table S1: Rum red deer (*Cervus elaphus*) linkage map after Build 5. Table is provided in file TableS1_CervusElaphus_Final_Linkage_Map.txt Column header are as follows:

SNP.Name	SNP Name
BTA.Chr	cattle chromosome
BTA.Position	cattle base pair position (BTA UMD v3.0)
CEL.LG	Deer linkage group
CEL.Order	Marker order on deer linkage group
cMPosition.SexAveraged	sex-averaged linkage map position (cM)
cMPosition.Female	female linkage map position (cM)
cMPosition.Male	male linkage map position (cM)
Skeleton.SNP	indicates if SNP is included in the skeleton map (see main text)
PAR	indicates if SNP is in the pseudoautosomal region
Estimated.Mb.Position	the estimated genomic position on the deer genome (see methods)
inf.mei	number of informative meioses
inf.mei.PK	number of informative meioses where grandparental phase was known
tot_f	number of informative meioses in females
tot_m	number of informative meioses in males
pk_f	number of informative meioses in females with phase known
pk_f	number of informative meioses in males with phase known
A1	major reference allele
A2	minor reference allele
CallRate	SNP call rate in original dataset (N_{IDS} = 2361)
Q.2	minor allele frequency
PseudoAutosomalSNP	indicates if sex-linked SNPs (CEL34) are in the pseudoautosomal region.

Table S2: Data for Figure S5, comparison of map positions between Cattle (bp, build vUMD 3.0), Deer (cM, Build 5) and Sheep (bp, build Oar_v3.1) for the X chromosome. Table is provided in file TableS2_X_Cattle_Cervus_Ovis.txt.

Table S3: Predicted approximate positions of unmapped SNP loci from Build 5. Table is provided in file TableS3_Predicted_Positions_of_Unmapped_Loci.txt. Column headers are as follows:

Window.Start	cM position of the start of the window of most likely position
Window.Stop	cM position of the end of the window
CEL.LG	Deer linkage group identifier
SNP.Start	First mapped SNP at the start cM position
SNP.Stop	Last mapped SNP at the end cM position
chunk	chunk identifier
SNP.Start.Of.Chromosome	Indicates if the most likely position is at the beginning of the chromosome
SNP.End.Of.Chromosome	Indicates if the most likely position is at the end of the chromosome
Unmap.SNP.vec	Vector of SNPs within the unmapped chunk

Table S4: Probabilities of crossing over within 1Mb windows in males and females. Table is provided in file TableS4_Recombination_Landscape_Info.txt. Column headers are as follows:

CEL.LG	Deer linkage group identifier
Window	Window order
Start	Mb position of the start of the window
Stop	Mb position of the end of the window
Locus.Count	Number of loci within the window
Mean.Inf.Count	Mean number of informative loci
cM	Sex-averaged recombination rate
cM.Male	Male recombination rate
cM.Female	Female recombination rate
Window.To.End	Window order from the other end of the chromosomes
FM.Rate	Ratio of female to male recombination rate
adj.cM	Sex-averaged recombination rate adjusted for chromosome size
adj.cM.Male	Male recombination rate adjusted for chromosome size
adj.cM.Female	Female recombination rate adjusted for chromosome size
adj.FM.Rate	Ratio of female to male recombination rate adjusted for chromosome size

Table S5: BLAST results for SNP flanking sequences in the Deer against the Sheep (Oar_v3.1) and Cattle (Btau_4.6.1) genomes in order to determine lineage of origin. Table is provided in file TableS5_BLAST_Lineage_Results.txt. Column headers are as follows:

Locus_Name	SNP ID
Species	Reference sequence, Ovis or Bos for sheep and cattle, respectively.
bit	Bit score of local alignment.
Chr	Reference species chromosome.
PCmatch	Percentage match between query and reference sequences.
matches	Number of matching bases between query and reference sequences.
mismatches	Number of mismatching bases between query and reference sequences.
gaps	Number of gaps between query and reference sequences.
SeqStart	Query sequence start position.
SeqSto	Query sequence stop position.
ChrStart	Reference sequence start position.
ChrStop	Reference sequence stop position.
eval	Expect value (E): the number of hits one can expect to see by chance when searching a data
Ncount	Number of unknown bases in the deer query sequence.
Informative.Length	Number of known bases in the deer query sequence.
PCmatch.Full	Percentage of matches at known bases.
Count	Number of independent hits for the query sequence.
BTA3Chr	Chromosome on cattle genome version vUMD3.0.
BTA3Position	Position on cattle genome version vUMD3.0.
CEL.LG	Deer linkage group.
cMPosition.run5	Deer build 5 centimorgan position.

Table S6: Raw data and results for binomial tests for the transmission distortion analysis. Alleles were assigned as A or B as the first and second reference allele in the GenABEL files. Table is provided in file TableS6_Transmission_Distortion.txt. Column headers are as follows:

A.Count	Number of A alleles transmitted from FID to offspring
SNP.Name	SNP ID
Parent	Indicates if FID was the father or mother
Geno.Count	Number of informative transmissions
P.val	P-value from the exact binomial test
CEL.order	The order of SNPs on the linkage group
CEL.LG	Linkage group
Fission	The fission/fusion history of the chromosomes
cMPosition.run5	Deer build 5 centimorgan position.
Dummy.Position	The estimated genomic position on the deer genome
Bin	1Mb Window in which the SNP falls
